# Comparative Proteomics of Mouse Tears and Saliva: Evidence from Large Protein Families for Functional Adaptation

**DOI:** 10.3390/proteomes3030283

**Published:** 2015-09-07

**Authors:** Robert C. Karn, Christina M. Laukaitis

**Affiliations:** College of Medicine, University of Arizona, Tucson, AZ 85724, USA; E-Mail: cmlaukai@email.arizona.edu

**Keywords:** mouse, tears, saliva, proteome, lacrimal gland, salivary glands, adaptation

## Abstract

We produced a tear proteome of the genome mouse, C57BL/6, that contained 139 different protein identifications: 110 from a two-dimensional (2D) gel with subsequent trypsin digestion, 19 from a one-dimensional (1D) gel with subsequent trypsin digestion and ten from a 1D gel with subsequent Asp-N digestion. We compared this tear proteome with a C57BL/6 mouse saliva proteome produced previously. Sixteen of the 139 tear proteins are shared between the two proteomes, including six proteins that combat microbial growth. Among the 123 other tear proteins, were members of four large protein families that have no counterparts in humans: Androgen-binding proteins (ABPs) with different members expressed in the two proteomes, Exocrine secreted peptides (ESPs) expressed exclusively in the tear proteome, major urinary proteins (MUPs) expressed in one or both proteomes and the mouse-specific Kallikreins (subfamily b KLKs) expressed exclusively in the saliva proteome. All four families have members with suggested roles in mouse communication, which may influence some aspect of reproductive behavior. We discuss this in the context of functional adaptation involving tear and saliva proteins in the secretions of mouse lacrimal and salivary glands, respectively.

## 1. Introduction

In the eyes of mammals, the corneal epithelium is continually kept wet by basal tears [1,2] that lubricate the eye and help to keep it clear of dust and other particles [3]. Other functions of tears include: (1) creating a smooth reflective optical surface; (2) supplying oxygen and nutrients to the cornea; (3) carrying waste products away from the cornea; (4) preventing bacterial growth on the ocular surface with antimicrobial substances and by providing access to white blood cells; and (5) flushing harmful bacteria and other microbes from the eye [3,4,5].

The lacrimal glands are the major sources of proteins in tears, although this fluid also contains water, glucose, salts and lipids secreted by a variety of sources [4,5,6]. While these components are common to the eyes of most or all mammals, other components, especially proteins, may be present in tears of some mammals but not others because they confer functions that are adaptive to the ecological niche of those possessing them [7]. Many of these specialized proteins have yet to be characterized and/or have as-yet-undescribed functions.

In the past 15 years, the rise of genomic and proteomic sciences has provided the opportunity to study the genes that are expressed to produce the array of proteins characteristic of a particular tissue. Proteomic studies represent a direct means of identifying the array of proteins expressed in a particular type of cell and/or in the fluid it secretes. The major advantage of such studies is that proteins identified at a high probability from two or more high quality peptides can be confidently believed to be present in the protein mixture analyzed.

The study we report here was originally conducted to determine the number of different androgen-binding protein (ABP) monomers and dimers that could be found in mouse tears. Both single dimension (SDS; 1D) and two dimension (IEF × SDS; 2D) gels were tested for the maximum number of ABP identifications obtainable from each. We also tested two different proteolytic enzymes, endopeptidase Asp-N (hereinafter Asp-N) and the more traditional trypsin, in the LC-MS/MS analysis because *in silico* analyses suggested that Asp-N might produce fewer and larger ABP peptides. As the study progressed, we realized that these variations could also produce a mouse tear proteome, which would be the first reported based on literature searches we conducted during the study. Therefore, the first objective of the project we report here was to obtain a tear proteome of the genome mouse (C57BL/6) from proteins separated in the various kinds of gels described above and digested either with Asp-N or trypsin before identification by LC-MS/MS analysis.

Dysfunction of the lacrimal glands can be either congenital or acquired, but generally results in lack of appropriate tear production, which exposes the eyes to damage from microbes and microparticles. Inherited abnormalities of lacrimal gland development can lead to the absence of glandular structures, while nervous system dysfunction can interfere with the stimulation of glandular secretion [8]. These disorders tend to affect both lacrimal and salivary glands. Autoimmune diseases, such as Sjogren syndrome, can attack one or both glands, decreasing their ability to produce tears and saliva [9]. Thus, we were interested in identifying both the unique and the shared secreted components of lacrimal and salivary glands in normal mice.

Since the creation of the first inbred laboratory mouse strains by Little (see [10]) and, later, Strong [11], the mouse has been widely used as an experimental organism in studies of human pathological conditions. There are several mouse models for Sjogren syndrome that recapitulate the dry mouth and dry eyes in the human disease [12], and it would therefore be useful to have both the tear and saliva proteomes of normal mice for comparison with these models.

We recently produced the saliva proteome of the genome mouse (C57BL/6) and the genome rat (BN/SsNHsd/Mcwi) using multidimensional protein identification technology (MUDPIT) for the purpose of studying rapidly evolving proteins and their genes [13,14]. In the original study, we focused on the independent expansions of the mouse and rat kallikrein subfamilies expressed in saliva and asked how selection influenced their evolution [14]. Here, we report the first tear proteome in an inbred strain of laboratory mouse, the genome mouse C57BL/6. We present a comparison of mouse tear proteins with saliva proteins, which should allow us to identify those shared and unique to each secretion, and which may in turn help shed light on their functions.

Because laboratory mice have been widely used as experimental organisms in studies of human pathological conditions, it is important to understand the ways in which mouse physiology is comparable to human physiology and the ways in which it is not. The discovery and description of three large protein families that influence mouse communication have arisen from our studies of mouse Androgen-Binding Proteins (ABPs; [15]), studies of Exocrine Secreted Peptides (ESPs; [16]) and studies of Major Urinary Proteins (MUPs; [17,18]) by other laboratories. We show that these mouse protein families are represented to varying degrees in the mouse tear proteome (this report) and the mouse saliva proteome [13,14], and yet the studies cited above have shown that these protein families have no counterparts in humans. We discuss the possibility that some of the proteins unique to each secretion represent important adaptations of the mouse tear and saliva protein complements, especially with respect to mouse communication.

In summary, our goals in this project were two-fold: (1) To obtain a tear proteome of the genome mouse (C57BL/6), using both single-dimension (1D) and two-dimension (2D) gel separations of tear proteins; and (2) To compare the tear proteome with the previously reported saliva proteome of that mouse strain, determining which proteins are shared and which are unique to each secretion.

## 2. Experimental Section

### 2.1. Tear Fluid Sources and Treatment

We obtained six male and six female mice of strain C57BL/6 from Jackson Laboratory (Bar Harbor, ME, USA) and treated them in accordance with University of Arizona Institutional Animal Care and Use Committee procedures under protocol 08-138. We anesthetized them as previously described [19] and stimulated lacrimation with intraperitoneal injection of 15 ng of pilocarpine in normal saline (Sigma, St. Louis, MO, USA; [20,21]). Tear fluid was collected by pipetting a 10 µL drop of sterile normal saline at the edge of the eyelid, waiting 5 s and removing the fluid with the micropipetter [21]. The process was repeated four more times such that 50 µL of eyewash was obtained from each animal. The animals of this inbred strain are essentially clones and thus the eyewashes from animals of each sex were pooled separately for analysis (300 µL total for each gender). We dialyzed the fluid against 100 mM ammonium bicarbonate buffer, pH 8.3, reduced the volume ten-fold in a Speed-Vac and dialyzed a second time against the same buffer.

### 2.2. Proteomics Analysis

**Figure 1:**
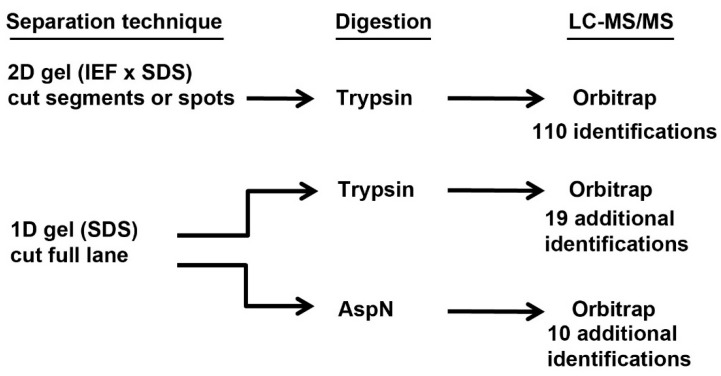
Flow chart for two-dimensional (2D) and one-dimensional (1D) gel experiments.

Karn *et al.* [21] reported the materials and LC-MS/MS methods in a previous study of C57BL/6 mouse tear ABPs and so they will be summarized only briefly here. The mouse saliva proteome was previously published [13,14]. We provided the dialyzed and concentrated tear fluids to the University of Arizona Proteomics Core Facility where personnel determined the protein quantity in the samples and performed single-dimension (1D) SDS gel separations and two-dimension (2D; isoelectric focusing × SDS gel electrophoresis) gel separations of equal protein quantities for each gender. A flow chart of these three experiments is shown in Figure 1. We performed gel separation prior to digestion because the original purpose of the experiment was to identify the subunits associated in individual ABP dimers in mouse tears, whereas the subunits of any dimer would have been confused with subunits of others by in-solution digestion. Gel pieces to be analyzed were excised in the Proteomics Core laboratory, chopped into 1 mm^3^ pieces, destained, reduced and alkylated, and enzymatically digested before LC-MS/MS analysis. In the case of the 2D gels, six regions of major protein staining pattern were excised. Together, all six regions constituted the entire portion of the gel that contained proteins based on staining.

The proteins in a complete lane of a 1D gel were digested with trypsin or with Asp-N (Figure 1). The proteins in each 2D gel piece were digested with trypsin (Figure 1). We digested proteins in the gel pieces [22] because it is an accepted technique that is widely used and is the standard procedure in our Core facility. We recognize that there are other methods of digesting gel-separated proteins with a variety of benefits and pitfalls [23]. LC-MS/MS analysis of the digested proteins was carried out using a LTQ Orbitrap Velos mass spectrometer [21].

### 2.3. Protein Identification from Proteomic Data

Data-dependent scanning was performed by Xcalibur v 2.1.0 [24] using a survey mass scan at 60,000 resolution in the Orbitrap analyzer, scanning *m*/*z* 400–1600, followed by collision-induced dissociation tandem mass spectrometry (MS/MS) of the most intense ions in the linear ion trap analyzer. Dynamic exclusion was used to minimize masking of low-abundance peptides by the more abundant ones. MS/MS spectra were searched against Uniprot *Mus musculus*, appended with ABP sequences provided by the author (Karn), using Thermo Proteome Discoverer 1.3 (Thermo-Fisher, Waltham, MA, USA). Proteins were identified at 99% confidence with XCorr score cut-offs [25] as determined by a reversed database search. The results were displayed with Scaffold v 3.6.1 (Proteome Software Inc., Portland, USA), a program that relies on various search engine results (Sequest, X!Tandem, MASCOT) and uses Bayesian statistics to reliably identify more spectra [26,27]. Scaffold was used to validate MS/MS-based peptide and protein identifications with criteria described in [21].

## 3. Results and Discussion

### 3.1. Protein Identifications in Tears Made under Three Different Conditions

In our initial experiments, tear proteins of both sexes of C57BL/6 mice were separated on an SDS gel (Supplementary Materials, Figure S1). The entire lane from each gender was excised and the proteins digested with either Asp-N or trypsin before LC-MS/MS analysis (Figure 1). These experiments were the single-dimension gels and their protein reports are shown in Table S1 and Table S2. In later experiments, tear proteins of both sexes of C57BL/6 mice were separated on 2D gels and the protein-stained region of each was cut into six pieces (see Supplemental File 5 in reference [21]). The proteins in these pieces of the 2D gels were digested with trypsin before LC-MS/MS analysis (Figure 1); see Table S3 and Table S4 for protein reports. The proteins identified in all of these experiments are summarized in a comprehensive table (Table S5). The optimum identification of ABP subunits was obtained from 2D gel separation, followed by trypsin digestion and LC-MS/MS analysis ([21]; also described below).

In all, 152 different proteins were identified by the combination of the results of all three different treatments. Converting IPI accession numbers to Uniprot numbers eliminated 13 duplicate identifications from our list, reducing the final number to 139 different proteins (Table S5). Most of these (110/139; 79%) were obtained from the 2D gel experiment, however, the 1D gel with trypsin digestion produced an additional 19 identifications (14%) and the 1D gel with Asp-N digestion produced an additional ten identifications (7%). It is possible that some proteins found in 1D gel lanes were not identified in the 2D gels because there was loss of some proteins either in the IEF dimension of the 2D system or in the region that appeared not to contain proteins and was ignored.

Clearly, trypsin digestion prior to LC-MS/MS was a more efficient means of identifying proteins overall than was Asp-N (61 *vs.* 19 in 1D gels; data consolidated in Table S5 from Table S1 and Table S2 with IPI numbers changed to Uniprot and duplicates removed). This can probably be attributed to the high level of efficiency of trypsin's endopeptidase activity. Asp-N digestion was particularly disappointing compared to trypsin’s ability to produce identifiable ABPs. The least identifications were obtained from a single SDS gel electrophoresis lane digested with Asp-N, it identified only A20 and BG2. However, Asp-N digestion apparently produced 19 unique peptides not produced by trypsin digestion probably because Asp-N cleaves proteins on the N-terminal side of Asp, whereas trypsin cleaves on the C-terminal side of Arg and Lys. That conclusion is supported by the observation that 10/19 proteins (53%) found in the Asp-N analysis were unique to that experiment whereas 20/61 (33%) were unique to the trypsin 1D analysis.

The 2D gel experiments identified almost twice as many proteins as the 1D SDS gel experiment that was followed by trypsin digestion (110 *vs.* 61), including more ABP proteins (13 *vs.* 6). In their recent study of 2D gel proteomic analysis and PCR/qPCR transcriptome analysis of ABP proteins in tears, Karn *et al*., [21] observed that every protein identified in the 2D gel proteome analysis had a corresponding transcript in the transcriptome analysis. Some ABP transcripts were identified that did not have a corresponding protein, however, qPCR showed that these were expressed 2–5 orders of magnitude below those with corresponding proteins (Supplemental File 9 in reference [21]). Their analysis suggests that, at least in the case of the ABP constituents of mouse tears, the 2D gel-based proteome analysis must have identified all or nearly all the ABP proteins.

We employed conservative identification criteria in all three experiments and so we expect that some proteins may have been missed on that account. We note that 50% of the proteins in Table S5 (39 in male tears and 30 in female tears) were identified in one sex but not the other. While our criteria were chosen to produce a very high probability that any identification was real, they also may have caused some proteins to appear to be sex-limited in their expression when in actuality they are not. We feel that it is far more likely that examples such as ABPA3 and ABPBG3 (5/6 male 2D pieces of each were positive; all female pieces were negative) are sex-limited in their expressions. Examples such as 14-3-3 protein sigma and cathepsin L1 (1/6 male 2D pieces of each were positive; all female pieces were negative) must be treated with caution. It is important to note that individual identifications in one sex are reliable under these criteria but that absence in the other sex (*i.e.*, negative data) is not.

### 3.2. Proteins in Mouse Tear and Saliva Proteomes

**Figure 2:**
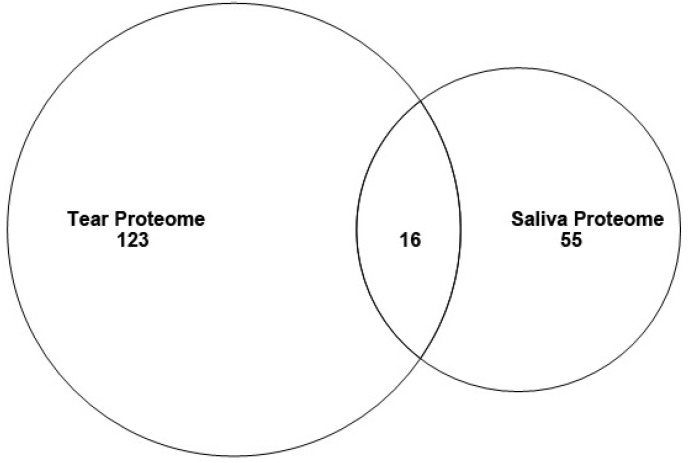
Venn diagram showing overlap between proteins identified in the saliva and tear proteomes.

The technology used in this study was different from the multidimensional protein identification technology (MUDPIT) that we used in the mouse saliva proteome [13,14]. The most important difference is that the tear proteome results we report here benefited from the more accurate Orbitrap analysis. The 139 different proteins that were identified in our mouse tear proteome number nearly twice as many as the 71 that we reported for a mouse saliva proteome (Figure 2, this report) [13,14]. Although both of these proteomes have fewer proteins than many others reported, it is our feeling that a deeper proteome is not always a better proteome. In our earlier analysis, we compared the results of two human saliva proteomes, one of which reported ten times more identifications than the other and we showed that nearly 2/3 (569/885) of the proteins found in the more extensive proteome lacked a signal peptide [13]. Thus, the deeper proteome may reveal less highly-represented proteins, but at the expense of detecting more contaminating intracellular proteins.

Table 1 shows the 16 of these that are shared between the two proteomes, *i.e.*, 12% of the tear proteins and 23% of the saliva proteins in those proteomes, respectively (Figure 2). Thus, the majority of proteins in each are unique to that secretion.

Previous analysis of two human saliva proteomes of very different depth identified a surprising number of proteins without signal peptides in the deeper one [13], suggesting the presence of contamination from cellular breakdown. We expected the proteins shared between saliva and tears to be enriched for such intracellular proteins, however only one (Titin isoform N2-A) of the 16 proteins found in Table 1 is intracellular. The other proteins shared between the two tissues likely reflect the overlapping functions of the secretions. The protective enzymes Carbonic anhydrase, DNAse1, Kallikrein-1, Lactoperoxidase and Trypsinogen 7 are all capable of combating microbial growth as is the polymeric immunoglobulin receptor, which is important in immunity. Keratin intermediate filament 16a and Cytokeratin 5 are likely shed from the extracellular matrix surrounding the cells lining the oral and orbital cavities and reflect their importance in maintaining tissue integrity.

**Table 1:** Shared Tear and Saliva Proteins.

Protein Name	Uniprot Number
16.5 kDa submandibular gland glycoprotein	Q9DA65
Carbonic anhydrase 6	P18761
Deoxyribonuclease-1	P49183
Kallikrein-1	P15947
Keratin intermediate filament 16a	Q9EQD6
Keratin, type II cytoskeletal 5	Q922U2
Lactoperoxidase	Q91WA0
Major urinary protein 5	P11591
Nucleobindin-2	P81117
Polymeric immunoglobulin receptor	O70570
Prolactin-inducible protein homolog	P02816
Protein LEG1 homolog (Mus musculus)	Q8C6C9
Serum albumin	P07724
Titin isoform N2-A (fragment in saliva)	E9Q8N1
Trypsinogen 7	Q9CPN9
WAP four-disulfide core domain protein 12	Q9JHY3

### 3.3. Major Protein Groups in Mouse Tears and in Mouse Saliva

Most of the proteins in each proteome were unique in that no other similar proteins from a particular family were also found. There were, however, larger or smaller groups of proteins representing families found in each proteome. For example, both proteomes contained representatives of the large mouse androgen-binding protein (ABP) family, thirteen in tears and three in saliva. It is striking, though, that the ABP proteins found in each secretion differ entirely [21]. We found six exocrine-secreted peptides (ESPs) in tears but none in saliva. The lipocalin family was represented by two MUPs (MUP2 and MUP4) unique to tears and MUP5 that was shared by both tears and saliva. Additionally representing lipocalins were two odorant-binding proteins (OBPs) unique to tears and one unique to saliva, as well as four other lipocalins found only in tears. Twelve unique keratins were found in tears but none were unique to saliva, although the two proteomes shared two other keratins (Table 1). Finally, the two proteomes shared Kallikrein-1, but only saliva contained representatives from the mouse-specific subfamily b; thirteen reported by Karn and Laukaitis [14].

It is not surprising that we found more keratin proteins in tears than saliva because of the close association between tears and the corneal surface. A similar association of unique proteins is found in saliva, where proline-rich proteins are enriched in the oral cavity because they bind tannins and maintain dental surfaces, as are cysteine-rich proteins that retard the growth of oral flora (reviewed in [28]). The remaining three groups of unique proteins in tears have been implicated in mouse communication: the ABPs [29], ESPs [16,30,31], and MUPs [32]. The presence of multiple ESPs reassures us that they are identifiable by standard proteomic techniques and were not lost due to their small size in MUDPIT processing of salivas, as feared previously [14].

Very likely, different investigators will be interested in different groups of proteins in these secretion proteomes, however it is striking that there is such a high representation of three groups of proteins in mouse tears that have been implicated in communication. The ABPs, the ESPs and the MUPs all represent protein families that have undergone significant expansions in the mouse genome [15,16,17,18,33]. We have also speculated that the mouse-specific kallikrein subfamily b and rat-specific kallikrein subfamily c could have similar roles in communication given their sex-limited expression and lineage-specific expansion patterns [14].

While it is not our intention to extensively compare these two mouse proteomes to their human counterparts, we would be remiss if we did not point out that humans do not have specialized protein groups corresponding to mouse ABPs, ESPs, MUPs, or the rodent-specific kallikrein subfamilies. We suggest that these rodent-unique protein groups, as expressed in tears and saliva, represent the footprints of functional adaptation (see below). Such major differences in protein constellations between rodents and humans must be considered in studies using mouse as a model for human disease.

### 3.4. Posttranslational Modifications of Proteins in Tears

**Figure 3:**
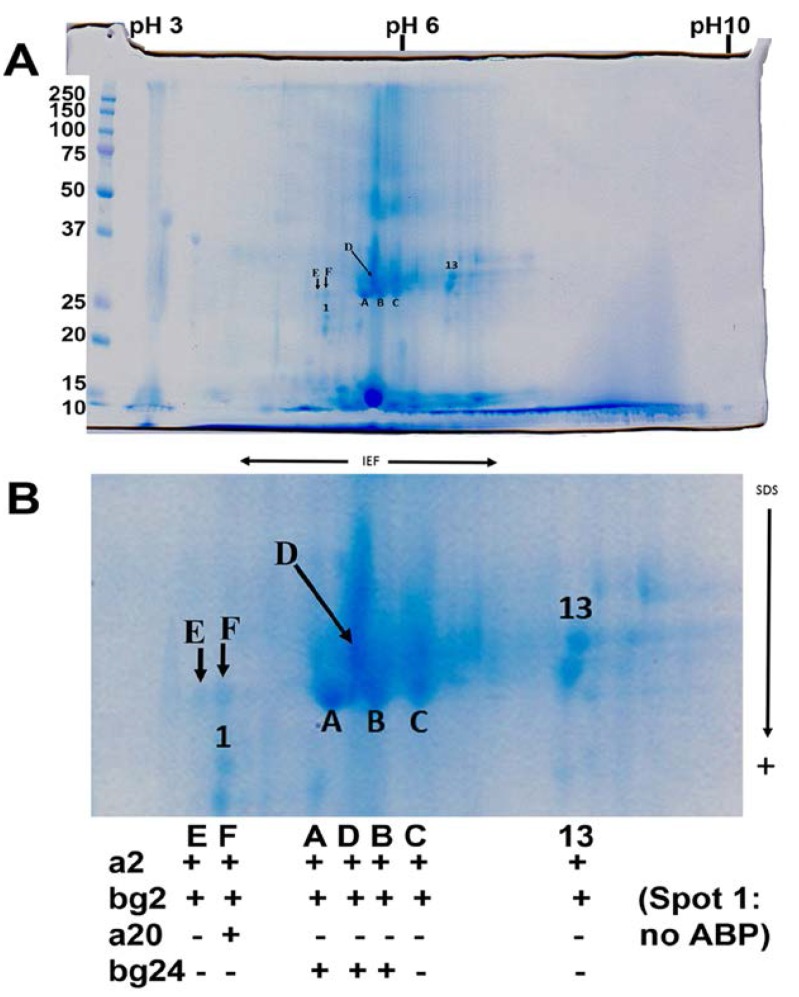
Two-dimensional gel (**A**), with center enlarged (**B**) showing excised spots and their ABP subunit content. The spots were sampled across a region of divergent isoelectric points but very similar molecular weights and these were subsequently excised and digested with trypsin for analysis by LC-MS/MS (see results in Figure 4). A + means the subunit was present, and a – means it was absent.

In designing this study, one of our hopes was that excision of protein spots from 2D gels, followed by digestion and LC-MS/MS analysis would allow identification of ABP dimers with different combinations of alpha and beta-gamma subunits, dimeric quaternary structures that are stabilized by disulfide bridging (reviewed in [34]). Two such ABP dimers in saliva have been identified, one an A27:BG26 and the other an A27:BG27. We performed a limited number of excisions of spots from a 2D gel of male tears and found A2 and BG2 subunits in seven spots arrayed linearly across the IEF dimension in the center of the gel, such that they had different isoelectric points but the same or nearly the same molecular weights (Figure 3). One of the spots also yielded an A20 subunit, suggesting that it might have contained A20:BG2 dimers as well as A2:BG2 dimers. Three other spots contained BG24 subunits, suggesting that they also contained A2:BG24 dimers in addition to A2:BG2 dimers.

**Figure 4:**
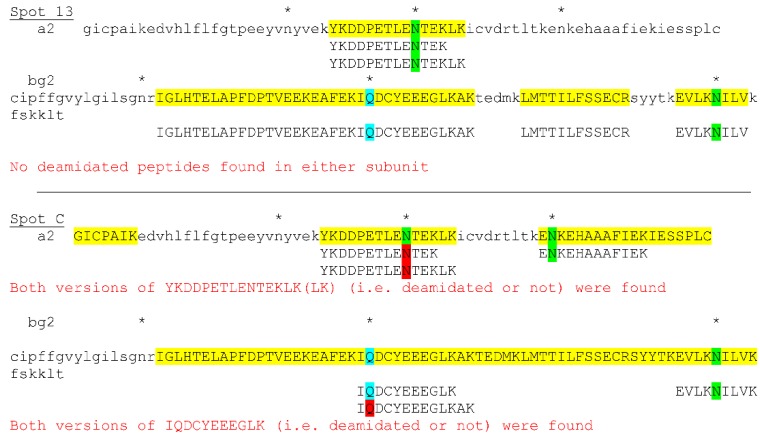
Examples of excised spots showing ABPA2 and ABPBG2 subunits with various Asn (green highlighting) and Gln (blue highlighting) residues some of which have been deamidated (red highlighting). Asterisks identify deamidatable Asn and Gln residues. Capital letters with (yellow highlighting) identify peptides found. See Figure S2 for the entire analysis and Table S8 for *Abp* coordinates and accession numbers.

The possible presence of as many as seven A2:BG2 dimers with variable isoelectric points could be explained by deamidation if there are sufficient Asn and Gln residues collectively in the A2 and BG2 subunits. There are three Asn residues in A2 and two in BG2. There is also a Gln residue in BG2, making a total of six potentially deamidatable residues in the dimer. Theoretically, there could be as many as seven forms with various combinations of deamidated residues, including one without any deamidation at all. We analyzed the peptides produced by LC-MS/MS analysis, which revealed some peptides with deamidated residues, however, not all peptides were identified (Figure 4, spot C). At this time we can only say that there were multiple forms of A2:BG2 identifiable as individual spots on the gel in the IEF dimension and that some had more than one residue deamidated (e.g., spot B in Figure S2). We did not pursue the excision of more spots because LC-MS/MS analysis of so many individual spots proved to be prohibitively expensive.

### 3.5. Comparative Proteomics and Adaptation of Two Exocrine Secretions, Tears and Saliva

Other studies have shown that genes involved in adaptation and functional innovation often show the footprints of positive selection in elevated ratios of nonsynonymous to synonymous nucleotide substitutions (dN/dS; sometimes reported as the rate Ka/Ks; [35]) in their coding regions [36,37,38,39,40]. Their evolutionary histories also show evidence of frequent duplication, deletion and pseudogene formation [41,42]. Prevalent among rapidly evolving genes are those involved in immunity, reproduction, chemosensation and toxin metabolism [41].

In addition to showing higher rates of evolution and higher rates of duplication and loss, candidate reproductive proteins also often show sex-limited expression, an instance in which only one sex expresses a protein or where there is a large disparity in the quantity of the protein expressed by the two sexes. The cause may be obvious for those genes whose protein products are synthesized in organs and glands found in only one of the two sexes, however, one seldom associates sex-limited expression with exocrine glands such as the lacrimal and salivary glands.

The mouse tear and saliva proteomes are notably different from their corresponding human proteomes in the presence of members of large protein families, the ABPs the ESPs, the MUPs and the rodent-specific kallikrein subfamilies that have no counterparts in humans. The salivary ABPs mediate assortative mate selection based on subspecies recognition. The result is to limit gene exchange between subspecies where they meet [43,44], reviewed in [29]. There is evidence that, in doing so, ABP comprises a system of incipient reinforcement where subspecies make secondary contact at the house mouse hybrid zone in Europe [45]. A recent study has presented evidence that the *Abp* genes expressed in mouse tears are a paralog group completely different than the smaller group expressed in saliva and that this may be due to subfunctionalization following duplication in *Abp* gene history [21].

The ESPs are a large family of proteins with at least some members serving as rodent proteinaceous pheromones [16,30,31,46,47]. Female mice respond to direct facial exposure to an ESP that is expressed in male exorbital lacrimal glands and released into tear fluid. The response is up-regulation of *c-Fos* and *Egr1* gene expression in vomeronasal sensory neurons. Mouse ESP1 appears to enhance a female sexual receptive behavior, lordosis, upon male mounting and copulation [47] and ESP22 was found to be released specifically in juvenile tear fluids and to inhibit the sexual behavior of adult male mice [46].

The MUPs are a family of lipocalins that mediate female recognition of potential mates (reviewed in [32]). Each adult mouse expresses a pattern of 8–14 different MUP isoforms in its urine, which is determined by its genotype and by its sex because some *Mup* genes show sex-limited expression [32]. MUPs have also been implicated in male-male aggression [48,49] and other studies have shown that both MUPs [50], and a hypothetical MUP peptide formed from the six N-terminal residues EEARSM [51,52], are androgen-regulated nonvolatile compounds capable of accelerating puberty in female mice.

Mice and rats each have a species-specific subfamily of kallikreins expressed in saliva, called subfamily b in mice and subfamily c in rats. Karn and Laukaitis [14] reported that twelve of the male sex-limited expressions in the mouse saliva proteome were members of the *Klk1b* mouse subfamily. Only *Klk1* (evolutionarily basal to mouse subfamily b) and *Klk1b5* showed expression in both sexes and in both cases, there was substantially more expression in female saliva. It has been traditionally held that salivary kallikreins play a role in wound healing, however, Karn and Laukaitis [14] argued that wound healing may not be the only function of these proteins. To support that view, they produced molecular evolutionary data showing that paralogs in the mouse subfamily b kallikreins have undergone rapid evolution, suggesting that other possible functions such as immunity, reproduction, chemosensation and toxin metabolism should be considered. Given the large number of these proteins expressed solely in the saliva of males, the most likely function may be some aspect of communication involved in reproduction.

Our comparison of the expressions of these four large mouse protein subfamilies suggests that the different paralogs of ABPs expressed in tears and saliva may have roles in communication. There is already substantial direct evidence for this from behavioral testing with saliva targets from congenic strains [43,44,45,53], and strong indirect evidence for tear ABP expressions, including observations of significant sex-limited expression of many paralogs and molecular evolutionary data for rapid evolution of *Abpbg*s in tears [21] and saliva [54]. There are six *Esp*s expressed in tears but they do not include *Esp1* encoding the protein affecting lordosis or *Esp22*, found to be released specifically into juvenile tear fluids (we studied adult mice). Here we report no strong evidence for sex-limited expression of the six *Esp*s that we identified, thus, we can provide no additional evidence that one or more of them have a specific pheromonal function. We found three members of the extensive MUP family (MUPs 2, 4 and 5) in this tear proteome study but we found only MUP5 in the saliva proteome [13,14]. None of these MUPs appears to be sex-limited in its expression. KLK1, the protein evolutionarily basal to the KLK1B protein subfamily, was the only mouse kallikrein that appeared in both the tear and saliva proteomes. With the exception of *Klk1b5*, all the other *Klk1b* expressions we observed in the salivary proteome were sex-limited to males [14]. More studies of the ESPs we found in tears and of the few MUPs found in both saliva and tears will be necessary to determine their functions in those fluids.

## 4. Conclusions

The C57BL/6 mouse tear proteome we report here contains nearly twice as many different proteins as the mouse saliva proteome that we reported previously for the same strain [13]. The few shared proteins between these two proteomes (16 proteins, 12% of the tear total and 23% of the saliva total) appear to be heavily biased toward enzymes capable of combating microbial growth and a protein involved in immunity. Of the remaining 123 tear proteins, 22 (18%) are members of three large protein families with no corresponding protein families in humans: Androgen-binding proteins (ABPs), Exocrine secreted peptides (ESPs), major urinary proteins (MUPs). All three of these, as well as the saliva-expressed mouse KLK1B subfamily, have been suggested to have roles in mouse communication and to influence some aspect of reproductive behavior. We discuss this in the context of functional adaptation involving tear and saliva proteins in the secretions of mouse lacrimal and salivary glands respectively.

## Supplementary Materials

Supplementary File 1

Supplementary File 2
