# Supplementary material for: Comparative Proteomics of Mouse Tears and Saliva: Evidence from Large Protein Families for Functional Adaptation
Source: Proteomes. 2015 Sep 7;3(3):283–97. doi: 10.3390/proteomes3030283 (PMC5217377; doi:10.3390/proteomes3030283)
Supplement: Supplementary File 2 [file proteomes-03-00283-s002.pdf]

Supplementary Materials

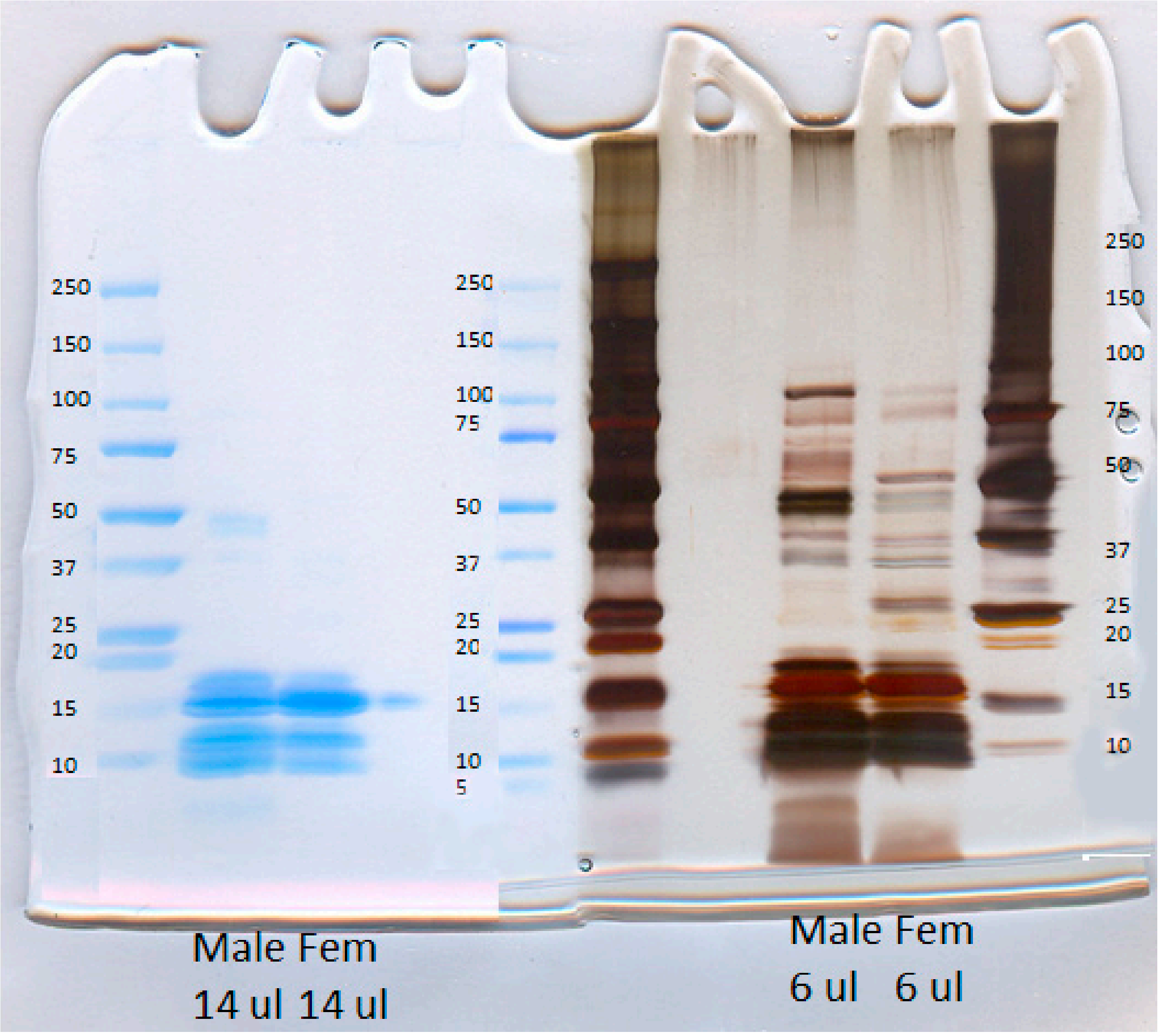

Figure S1. SDS one-dimension (1D) gel.

```
Spot 1      No ABP peptides found

Spot 13                       *             *               *
   A2   gicpaikedvhlflfgtpeeyvnyvekYKDDPETLENTEKLKicvdrtltkenkehaaafiekiessplc
                                   YKDDPETLENTEK
                                   YKDDPETLENTEKLK
                     *                        *                                     *
  BG2  cipffgvylgilsgnrIGLHTELAPFDPTVEEKEAFEKIQDCYEEEGLKAKtedmkLMTTILFSSECRsyytkEVLKNILVkfskklt
                       IGLHTELAPFDPTVEEKEAFEKIQDCYEEEGLKAK     LMTTILFSSECR     EVLKNILV

No deamidated peptides found in either subunit

Spot A                       *             *               *
   A2  gicpaikedvhlflfgtpeeyvnyvekYKDDPETLENTEKLKicvdrtltkenkehaaafiekIESSPLC
                                  YKDDPETLENTEK
                                  YKDDPETLENTEKLK
                     *                        *                                     *
  BG2  cipffgvylgilsgnriglhtelapfdptveekeafekIQDCYEEEGLKAKTEDMKLMTTILFSSECRsyytkevlknilvkfskklt
                                             IQDCYEEEGLK
                                             IQDCYEEEGLKAK
                                 *            *     *    *    *            *                  *
 BG24  cvpffagyagvisgsrlwlyhelsafngtpkETVAYEKIQDCYKEQGVKsqtlepqilasilvtpeclqyyseetftkikdalkkisqh
                                             IQDCYKEQGVK

Spot B                       *             *               *
   A2  gicpaikedvhlflfgtpeeyvnyvekYKDDPETLENTEKLKicvdrtltkENKEHAAAFIEKIESSPLC
                                  YKDDPETLENTEK            ENKEHAAAFIEKIESSPLC
                                  YKDDPETLENTEKLK
                     *                        *                                     *
  BG2  cipffgvylgilsgnrIGLHTELAPFDPTVEEKEAFEKIQDCYEEEGLKAKTEDMKLMTTILFSSECRsyytkEVLKNILVKfskklt
                                        EAFEKIQDCYEEEGLK
                                        EAFEKIQDCYEEEGLKAK
                                 *            *     *    *    *            *                  *
 BG24  cvpffagyagvisgsrlwlyhelsafngtpkETVAYEKIQDCYKEQGVKsqtlepqilasilvtpeclqyyseetftkikdalkkisqh
                                             IQDCYKEQGVK

 Spot C                       *             *               *
    A2  GICPAIKedvhlflfgtpeeyvnyvekYKDDPETLENTEKLKicvdrtltkENKEHAAAFIEKIESSPLC
                                   YKDDPETLENTEK            ENKEHAAAFIEK
                                   YKDDPETLENTEKLK
 Both versions of YKDDPETLENTEKLK(LK) (i.e. deamidated or not) were found
                      *                        *                                     *
   BG2  cipffgvylgilsgnrIGLHTELAPFDPTVEEKEAFEKIQDCYEEEGLKAKTEDMKLMTTILFSSECRSYYTKEVLKNILVKfskklt
                                              IQDCYEEEGLK                        EVLKNILVK
                                              IQDCYEEEGLKAK
 Both versions of IQDCYEEEGLK(AK) (i.e. deamidated or not) were found

 Spot D                       *             *               *
    A2  GICPAIKedvhlflfgtpeeyvnyvekYKDDPETLENTEKLKicvdrTLTKENKEHAAAFIEKIESSPLC
                                     DDPETLENTEK            ENKEHAAAFIEK
                                   YKDDPETLENTEK
                                   YKDDPETLENTEKLK  (only this one was deamidated)
                      *                        *                                     *
   BG2  cipffgvylgilsgnrIGLHTELAPFDPTVEEKEAFEKIQDCYEEEGLKAKTEDMKLMTTILFSSECRSYYTKEVLKnilvkfskklt
                                              IQDCYEEEGLK   (some were not deamidated)
                                              IQDCYEEEGLKAK (found both this and the one above it)
                                  *            *     *    *    *            *                  *
  BG24  cvpffagyagvisgsrlwlyhelsafngtpkETVAYEKIQDCYKEQGVKsqtlepqilasilvtpeclqyyseetftkikdalkkisqh
                                              IQDCYKEQGVK  (second Q was not deamidated)

 Spot E                       *             *               *
    A2  gicpaikedvhlflfgtpeeyvnyvekYKDDPETLENTEKlkicvdrtltkenkehaaafiekIESSPLC
                                   YKDDPETLENTEK
                      *                        *                                     *
   BG2  cipffgvylgilsgnrIGLHTELAPFDPTVEEKeafekIQDCYEEEGLKAKTEDMKLMTTILFSSECRsyytkevlknilvkfskklt
                                              IQDCYEEEGLK
                                              IQDCYEEEGLKAK
```

Figure S2. *Cont.*

```
Spot F                       *             *               *
   A2  gicpaikedvhlflfgtpeeyvnyvekYKDDPETLENTEKLKicvdrtltkENKEHAAAFIEKIESSPLC
                                  YKDDPETLENTEK           ENKEHAAAFIEK (not found deamidated)
                                  YKDDPETLENTEKLK  (both deamidated and not)
                     *                        *                                     *
  BG2  cipffgvylgilsgnrIGLHTELAPFDPTVEEKEAFEKIQDCYEEEGLKAKTEDMKLMTTILFSSECRsyytkevlknilvkfskklt
                                             IQDCYEEEGLK
                                             IQDCYEEEGLKAK   (no deamidated peptides found)
                     *           *         *     *
  A20  gicpaikedvrlflngtseeyveyvkQYKDDPVILENTAKIKQCVDSTLTEEDKIHATTFIEKIEASPIC
                                 QYKDDPVILENTAK
                                               IKQCVDSTLTEEDKIHATTFIEK

No deamidated peptides of A20 were found
```

Figure S2. Peptides found to be deamidated (and those not deamidated).Excised spots showing ABPA2 and ABPBG2 subunits have Asn (**green highlighting**) and Gln (**blue highlighting**) residues, some of which have been deamidated (**red highlighting**). Asterisks identify deamidatable Asn and Gln residues. Capital letters with (**yellow highlighting**) identify peptides found.
